# The bargaining of professionalism in emergency care practice: NHS paramedics and higher education

**DOI:** 10.1007/s10459-017-9802-1

**Published:** 2017-11-10

**Authors:** Assaf Givati, Chris Markham, Ken Street

**Affiliations:** School of Health Sciences and Social Work, University of Portsmouth, Portsmouth, UK

**Keywords:** Paramedic science, Higher education, Professionalization, Professionalism, Qualitative

## Abstract

Over the past 2 decades, as part of reforms to the National Health Service and with it organizational changes to ambulance work in the UK, paramedic education has undergone a process of academisation and a shift from in-house, apprenticeship weeks-long occupational training, to university-based undergraduate programs. While the professional regulation and standardization of Allied Health Professionals’ education in high-income countries has captured scholarly attention, the study of paramedic practice is still in its infancy and there is a need to explore its evolvement in relation to the fluid societal–political circumstances affecting its provision and demand. Based on interviews with front-line paramedics, paramedic educators and paramedic science students in the South of England, this article examines how the reforms to paramedic education have impacted the professionalization of paramedics and their discourse of professionalism. Framed within to the ‘new’ sociology of professions, the case of British paramedics demonstrates the complex nature of the relationship between the university and professional practice. It appears that universities, the providers of paramedic education, are caught between two opposing discourses of professionalism: on the one hand, that of providing a platform for students’ socialization and engagement with professionalism ‘from within’ practice which is based on students’ common goals and mutual experiences, and, on the other hand, serving as a conduit for managerial/organizational strategies of professionalism which appear to undermine the role of university socialization.

## Introduction

### The changing landscape of paramedic practice

Over the past couple of decades, as part of governments’ response to a host of demographic, economic, behavioral and societal variables affecting the health care landscape, the ambulance service underwent major changes to its education and practice. As Nancarrow and Borthwick (2005) point out, health professionals’ disciplinary boundaries are dynamic in nature and are defined in relation to fluid circumstances including societal expectations, cultural perceptions and technological advancements. Globally, the health care workforce is having to adapt to intensifying and unmet demand which, coupled with staffing shortages, is leading to the design of patient-centered care based consumers preferences by introducing professionalized and target-driven services (Allsop and Saks 2003; Freidson 2001; Nancarrow and Borthwick 2005). In order to enhance the scope of practice and public health roles of Allied Health Professionals (AHPs), which would allow for the expansion and enhancement of the health care workforce, there was a need to regulate their practice and expand their education and training programs. In the UK, new AHPs such as operating department practitioners, podiatrists, speech- language- , arts- and occupational therapists, clinical and biomedical scientists, and paramedics, followed the already-established health care occupations such as nursing, midwifery, radiography and physiotherapy, and entered universities, often as undergraduate programs (Timmons 2011; Woollard 2006).

In the case of paramedics, their role has typically been shifted since the 1970s from patient transport service to an increasingly professionalized and medicalized practice (Brooks et al. 2015). For example, Metz (1981), described ambulance work in the US during the late 1970s as a kind of “blue-collar profession“, a term used to capture the street-level, haphazard, unpredictable, often stressful encounters that are part of the ambulance work, which is rewarded with a high degree of professional autonomy yet with poor working conditions and low pay. However, over the past couple of decades, paramedic education and practice in countries such as the US, Canada, Australia and the UK has been transformed.

In Canada, the paramedic role has been adapted in order to meet the growing demand for emergency care services as well as the increasingly long waiting time for medical care and the ever-inflating costs of health care (Corman 2016). In Australia, paramedics developed greater medical skills as part of the solution for providing primary healthcare in small rural communities (O’Meara et al. 2012). Moreover, according to Williams et al. (2009), Australian paramedics extended their professional status by adopting a number of professionalization strategies including the development of a political alliance with the medical profession, regulating practice, and by developing training programs in Higher Education Institutions (HEIs).

As for the UK, for many years the ambulance service was restricted to the transportation of patients and was regarded as no more than low-paid, low-status manual labor (Kilner 2004). However, the introduction of reforms to the NHS (The Department of Health 1997) have included changes to ambulance service delivery and with it to paramedic education and training (Brooks et al. 2015), leading to the enhancement of paramedics’ medical skills and public health roles. It was only in 1966 that formal requirements for minimum level of training for ambulance staff in Britain was introduced (Ministry of Health 1966). The training was delivered ‘in-house’ by the ambulance service, which awarded certificates (Brooks et al. 2015). Following the 1974 NHS Registration Act, the ambulance service became part of the National Health Service (NHS), leading to an enhanced medicalization of ambulance work and to the emergence of *paramedic practice*, which increasingly included medical interventions (College of Paramedics 2014). Indeed, during the 1990s, ambulance services in the UK started providing complex medical procedures such as cardiac defibrillation, nebulization therapy and the administration of several prescription-only medicines (Craggs and Blaber 2008).

Parallel to these developments there is evidence that the demand for ambulance service in the UK, the cost of emergency care services and the job-strain on ambulance practitioners are all increasing significantly from year to year (National Institute of Health Research 2016). Whilst the driving forces behind these trends are not fully known, it appears to involve the impact of demographic changes and an ageing population, as well as organizational and practice-related changes to the already-complex system of unplanned care with its ambulance services, A&E departments, emergency mental health services, emergency telephone helpline and walk-in centers.

### Reforms to paramedic education in the UK

Central to the NHS modernization reforms, was the regulation of medical para-professionals, including paramedics, by a newly established umbrella regulator, the ‘Professions Supplemental to Medicine (CPSM)’, later named Health Professions Council (HPC). Paramedic practice underwent re-evaluation by an Audit Commission (1998), which outlined the enhancement of paramedics’ medical skills and professional responsibilities in order to enable them to *treat* and manage those patients with less serious conditions. Such changes to paramedic practice required reforms in the training programs and indeed the Audit Commission recommended that paramedic training should be delivered in universities (Brooks et al. 2015).

Upon regulation with the HPC and the introduction of standards of proficiency for paramedic practice (Health and Care Professions Council 2014), an accreditation procedure including benchmark curricular standards was developed by the College of Paramedics (2014), the professional body for paramedics and ambulance practitioners in the UK. Central to this cultural change management in the ambulance service was the “Bradley Report“, a strategic review of NHS Ambulance Service by the Department of Health (DoH 2005). The report emphasized the need to better-utilize practitioners’ skills in order to meet cost and efficiency targets, often referred to as a shift from “scoop and run“to “see and treat“.

From this point onwards, university education was the main route for registered paramedics, although it was still possible to become a student paramedic with the ambulance service and study while at work on the road. By 2017, there were 36 providers of paramedic education, of which 31 were universities. Of these programmers, 17 are offered at undergraduate (BSc) level. Furthermore, following a review of paramedic education (Lovegrove and Davis 2013), it was stipulated that from 2019 onwards BSc (Hons) will be the standard paramedic education pathway.

## Theoretical framework: higher education and the dual discourse of professionalism

Defining professional work has been an elusive and increasingly complex exercise for social scientists, due to the fast-changing environment in terms of cultural–organizational, societal and policy changes, global-international influences and the blurring borders between the public and private sectors (Evetts 2013; Noordegraaf 2007). The ‘classic’ approach to the study of professional work has been mainly in relation to *professionalization,* which is the process by which occupational groups obtain higher status and economic gains as well as the monopoly protection of the occupational jurisdiction, through *occupational closure* (Abbott 1988; Freidson 1986; Larson 1980; Parkin 1979; Witz 1990). Such occupational closure is achieved by restricting access to the occupational group’s knowledge and skills-base, typically through *credentialism*, which is the standardization of professional knowledge and the establishment of certification programs (Collins 1979, 1990), typically, in HEIs (MacDonald 1995). From an occupational closure view,

….the key to the definition of a profession remains the sheltered position of professions in the marketplace, with entry to the professions usually gained through obtaining relevant higher education credentials (Saks 2012, p. 4).

Occupational closure was effective in considering the *collective strategies* of occupational groups in their quest to enhance their economic and social standing and prestige. However, Evetts (2006, 2013) suggests that the preoccupation with closure strategies neglects the impact of the “internal” professional culture and the role of practitioners in shaping the profession ‘from within’ (Freidson 2001). This “new” sociology of professions recognizes the emergence of occupational strategies of professionalism which are generated *by practitioners themselves,* resulting from their ability to apply knowledge in practice, from their self-regulation and from their conduct, rather than from being “passive” members of a professional group (Cant et al. 2011; Noordegraaf 2007).

According to Freidson (2001), professionalism is the main organizing principle in service-related work such as AHPs. Professionalism is seen here as the formation of common practices and procedures that enable the negotiation of practice during interactions between members of the professional group and their consumers, as well as the production of a normative value system that is adopted by the individual practitioner (Evetts 2013). However, Freidson (2001) asserted that, for professionalism to be sustained, the occupational control of the work should be held by practitioners themselves, as they are the ones most familiar with complex work-related situations which require the utilization of expert knowledge and discretionary decision-making.

Therefore, from this perspective, the role of universities in shaping professions is not limited to the mere delivery of certificates and credentials that separate the professional from the non-professional. Rather, universities are conceptualized as a place whereby professional knowledge is constructed as part of practitioners’ academic journey and where shared professional characteristics are being formed. The university, like other large institutions (such as hospitals for example), is a place where professional identity and work-culture are constructed, as part of practitioners’ common interaction with vocational and educational experiences and as part of the shared challenges and collective values that are negotiated during a process of socialization in HEIs (Evetts 2013). Furthermore, the complexities and unpredictability of work-encounters call for specialist knowledge (that is obtained during education and training) as well as the exercise of discretionary decision making that warrants consumers’ trust in the practitioner (Evetts 2006).

Nevertheless, in recent years, professionalism has also been analyzed as a discourse of *occupational change and control* in service work organizations (Fournier 1999). When governmental and organizational budgets become scarce and consumers become more demanding, the state is trying to reshape the nature of professional work so that it is more economic, more commercial in nature and more entrepreneurial (Hanlon 1999). An example to this discourse is the way the government re-organized emergency care services in the UK in reply to demographic and economic pressures by regulating practitioners and by formalizing and medicalizing their training in HEIs. As a result of such market-driven managerialism, professionals have become

…part of large-scale organizational systems, with cost control; targets; indicators; quality models; and market mechanisms, prices, and competition (Noordegraaf 2007, p. 763).

In light of these tensions between managerial/organizational professionalism and occupational-level professionalism, we question the position of universities as part of AHPs professional projects. It appears that the external regulation of professional work, which has been labeled as “professionalization from above” (McClelland 1990), contradicts, and possibly undermines, the discourse of professionalism that is developed by practitioners themselves during university socialization. Moreover, it is possible that the standardization of expert knowledge in university, along with the newly-introduced regulatory measures and practice performance-indicators, threaten practitioners’ *professional autonomy* and discretionary decision-making that is founded on experience and on individual qualities (Jamous and Peloille 1970).

In their ethnographic study of the professionalization of paramedics in the UK, McCann et al. (2013) demonstrated the apparent tension that exists between the professional motivation of the senior, institutional level, and that of “street-level” paramedics. On the one hand, the management is looking to establish a discourse of “managerial professionalism” that is constructed by standardized procedures and emphasizes *practitioners’ accountability* based on adherence to institutional targets, external regulation, target-setting and performance review (Evetts 2013). On the other hand, street-level paramedics are seeking to sustain a discourse of “occupational professionalism” that is founded upon collegial authority, *practitioner’s discretion and trust*, and professional ethics. It appears as if universities are positioned at the heart of this tension. The university function both as standardizing agency that channels managerial aspirations, but also as a site for practitioners’ socialization, personal expression and an internalized academic journey.

## Methodology

This narrative-based qualitative research study was conducted during the academic year 2015/16, at a university providing Paramedic Science education in collaboration with the regional ambulance service in the South of England. Narrative-led research is an umbrella term that is designed to capture personal and human dimensions of experience over time in the context of the relationship between the individual experience and its social context (Connelly and Clandinin 2000). In practical terms, it is the systematic gathering, analysis, and representation of people’s own stories *as they tell them*. This approach allows analyzing the experiences as well as the meanings, values, beliefs and interpretations that the study participants attach to their lived experiences, and positioning them in relation to the theoretical framework guiding the investigation.

### The research setting

The UK Ambulance Service is currently operating in 13 regional trusts including a Scottish, Welsh, Northern-Irish and 10 English trusts. This research took place in the South of England, in a regional trust that has over 700 registered front line paramedics, as well as managers, team leaders, educators, clinical mentors and specialist paramedics such as air ambulance personnel. The university providing Paramedic Science education collaborates with the regional ambulance service and relies on it to provide clinical placement and clinical training to its students. Upon successful completion, students are eligible to apply for registration with the Health and Care Professions Council. Paramedics are in demand and employability rate, upon graduation, is near 100 per cent. At the time the study was conducted, the university program of studies was in the process of transition from foundation (FdSc) to BSc (Honors). The program, which has been delivered at the university since the academic year 2007/8, had approximately 75 registered students at the time that data was collected.

In order to reflect the views and experiences of those who shape paramedic education and practice ‘on the ground’, we selected members of the following participant-groups: front-line NHS paramedics from the regional ambulance service; NHS paramedic educators who are employed by the ambulance service to provide continuous professional development as well as support the practice elements of universities’ Paramedic Science programs; and Paramedic Science university lecturers who are employed by the university. The main data collection instrument was in-depth interviews with registered NHS paramedics (N = 20, including 10 women and 10 men; averaging 13 years of ambulance practice). In-depth interviews were chosen to enable a narrative-based approach that is led by interviewees’ own experience and interpretation of the social world (Coombes et al. 2008).

A secondary source of data collection was a focus group discussion (FGD) (N = 8) with students from one Paramedic Science program based at a university in the South of England. While the main focus of the investigation was to examine the impact of reforms to paramedic education on paramedics’ professional project and professional culture, as we discussed as part of the theoretical framework, it is important to also consider the role of university socialization in shaping professionalism. Therefore, FGD was set up to inspire participants’ “story-telling” in a group setting, which is not possible during one-to-one interviews (Bowling 2014). Moreover, considering that students may feel uncomfortable discussing their studies openly, we hoped that being part of a group will provide them with a degree of confidence and reassurance to express their views.

### Sampling, recruitment and ethical approval

We adopted *purposive* sampling strategy which is a deliberate, non-random sampling approach that is aimed at sampling a group of people who share particular characteristics that are central to the investigation (Bowling 2014). All of the participants in the in-depth interviews were registered paramedics at the regional ambulance service. Eight participants were “front-line” paramedics; six were from the education department of the ambulance service with which the university collaborates; and six were paramedic lecturers from the Paramedic Science host university. In order to recruit front-line paramedics, we initially approached the divisional manager at the regional ambulance service. The divisional manager approved paramedics’ participation and circulated the study information sheet via email to the regional ambulance team leaders. The team leaders then extended the invitation to their team members. It is possible that this indirect recruitment strategy limited the number of participants.

Paramedic educators from the ambulance service were approached both via email as well as in person during one of their periodic meetings with the academic team at the university. Lecturers from the academic team at the university (six out of seven) were approached in person at the university. Final year students attending the Paramedic Science program were invited to take part in a Focus Group Discussion both via the university email network as well as during one lecture. Eight out of thirty-five students agreed to participate in the study. Informed consent was obtained prior to all interviews and participants’ confidentiality was maintained throughout all stages of the investigation. All interviews were recorded and transcribed verbatim. Ethical approval was obtained on 16 June 2015 from the host university’s Science Faculty Ethics Committee (SFEC 2015-026).

### Data collection

Primarily data was obtained via in-depth interviews, which emphasize depth and richness of the data rather than its representativeness in a statistical sense (Bowling 2014). The use of open-ended and flexible questions as well as probing responses were considered to be able to provide better insight into interviewees‘views, opinions, experiences, and interpretations and, to a certain extent, to be able to represent the interviewees‘own language and bring their own voices to the fore (Byrne 2004). As argued by Gubrium and Holstein (2002), understanding social phenomena, social behavior and social organization is central to qualitative research, suggesting that the interview process should become more “democratized”. That is, rather than an “expert researcher” defining and refining the interview process, this is done directly by the participants who are in effect the “experts” in the social phenomena and the field in which they are nested. The role of the researchers is to enable participants to express themselves effectively and openly so that they inform the research questions, followed by systematic process of data analysis.

Prior to the interview, the study participants were introduced to the study information sheet and signed informed consent. During the interview they were asked pre-defined, broad questions that were derived from a review of the literature and, from the conceptual framework. The interview process was designed to obtain wealth and depth of data with only limited constraining of participants’ story-telling, while the main analytical work is done during the data analysis stage. Front-line paramedics were asked to describe their practice biography, reflect on their paramedic education, compare paramedic practice when they joined the ambulance service to current practice, discuss challenges to practice over time, and consider the nature of the paramedic workforce and of the paramedic professional culture. Often, they were asked to provide examples from practice or reflect on aspects of practice raised in their narratives.

Paramedic educators and academics were asked to consider similar topics as well as describe changes over time and challenges to paramedic education. As for the FGD, here too participants read the study information sheet prior to the interview and signed informed consent. Here, the focus was on the education/academic journey of the participants and the circumstances and factors shaping this experience. Interviewees were asked to describe their motivation for joining the academic program, reflect on the academic and the practical elements of the course and on their interaction with front line paramedics and with paramedic educators, as well as describe their aspirations and how they see their professional future and consider challenges to education and practice.

### Data analysis

Data analysis was conducted according to qualitative content analysis (QCA) (Mayring 2000; Schreier 2014), a method used to systematically describe the meanings of qualitative data. It is a procedure for analyzing extensive textual materials from various resources, including interview data, while reducing the material at hand so that it corresponds with the research question. Although in-depth interviews are designed to promote participants’ self-reflection and rich story-telling, and therefore often allow interviewees’ narratives to “wander” to areas beyond the research interest, the process of QCA provides a systematic and pragmatic approach to manage the data. The first stage of the data analysis is to read through the text and remove material that is strikingly unrelated to the research question. The next stage is to organize the text under pre-defined “contextual categories”, which are developed from the theoretical models informing the investigation and against which the data is considered. Textual material is copied and pasted under matching contextual categories. In our case, the following contextual categories/units were used to organize the text at this stage: professionalization; credentialism; “academic” versus “hands-on” knowledge in paramedic education; managerial professionalism; and occupational professionalism.

Although presented in advance, the contextual categories only serve to frame the analyzed text and ensure that it is considered in relation to the theoretical framework, while removing parts of the narratives that are not developing the investigation. These categories are repeatedly re-visited as data emerges, and, if the emerging interview-narratives present new research-related concepts and “leads” that were not previously considered, are modified accordingly. Through this dynamic approach, the inherently inductive character of QCA is safeguarded. Once this stage is completed, the researchers continue analyzing the text under each category to identify *emerging sub*-*themes* within the context of the original categories. Effectively, the method of QCA identifies certain key categories from the available research literature and from theoretical models and then turns to the data to consider how these categories or terms are manifested. In order to enhance the likelihood of analytical generalization, textual material is analyzed systematically to the point of saturation of sub-themes (Polit and Beck 2010).

## Findings

Participants’ narratives illustrate a number of important challenges to paramedics’ professional culture, in light of the reforms to paramedic education: the tension between the value of practice-experience and personality traits, and the increasing emphasis on the acquisition of academic knowledge in HEIs; the friction between the experienced paramedics (and ambulance technicians)^1^ who were trained prior to the educational reforms, and the “knowledgeable yet often inexperienced” university graduates; the perceived loss of the important worksite socialization, which is paralleled with the increased individualization and perceived “practice/practitioner isolation” that followed the reforms; and an assertion that university education opened greater job-opportunities to the new graduates, yet, may have a negative impact on job-retention and on the sustainability of the paramedic workforce.

### First theme: the academic framing of paramedic expert knowledge

Our analysis of participants’ narratives centers around three inter-related thematic strands, the first of which relates to the process of re-locating professional training from its previous “in-house” occupational setting to HEIs. The process of formalizing professional training as part of professionalization strategies has been linked with the enhancement of practitioners’ reputation and external legitimacy, but also with greater transparency of expert knowledge, greater external scrutiny and a reduction in practitioner’s professional discretion and autonomy (Jamous and Peloille 1970; Witz 1990). For the”pre-reforms”, experienced paramedics, the transition in education and practice was recognized. They had to adapt simultaneously to rapidly increasing demand for emergency care services, to growing job intensity, as well as to a newly-defined paramedic role. SK, the course leader of the BSc (Hons) Paramedic Science, reflects on changes to ambulance work over the past few years:

The first thing that you notice when you go back after being away from it for a while is just the intensity of it all. The fact that there is no downtime, there is no opportunity to catch your breath really. You’re off and then you go from one patient to the next with very little break in between. […] There are lots of tired, fatigued people out there doing this job. The intensity of it must be completely draining.

Similarly, PJ, has seen a dramatic change to the paramedic shift work since she qualified as a paramedic in 2009. In her narrative, she describes how it changed:

Back in 2009 when I started, I remember going for two days in a row without doing a job. Now you can do ten jobs in a day; there’s jobs just stacking and stacking and stacking…. It just never stops.

The new academic programs were designed to ensure that graduates are able to diagnose and deliver complex medical interventions. The BSc (Hons) Paramedic Science program (360 academic credits in total obtained over 3 years of study and clinical placement) includes the teaching of several biomedical subjects: anatomy and physiology (20 credits); the principles of evidence based practice (20 credits) and of scientific research (20 credits); conducting a final year research project (40 credits); study the principles of physical and biomedical sciences (20 credits) and pathological processes (20 credits). Furthermore, students also obtain the academic skills that are part of any undergraduate (Honors) program of study, alongside the “purely clinical” units and clinical placement. This has been a complete overhaul of the previous weeks-long courses, whereby practitioners had to “learn as they went along”. CJ, who attended the pre-reforms training, joined the London Ambulance Service in 1995. She recalls her first day in practice:

There I was…I was chucked out and it was literally sink or swim. […] we’ve given our own personal paramedic bag and it was literally thrown at you this bag. It was wrapped in plastic, it had a laryngoscope and stuff inside and I didn’t know what to do with this equipment. It was quite horrific time now that I think about it….”baptism of fire”.

For those paramedics who had been trained prior to the introduction of the academic programs, the educational and practice reforms were perceived as a gap in academic and practical knowledge while having to adapt to the expansion of their previous paramedic role. University programs, and their “product”, paramedic graduates, were often perceived by the veteran ambulance practitioners with mistrust and with cynicism. BT, for example, was an ambulance technician before eventually enrolling to Paramedic Science university program. Looking back, she recalls her concerns about the academic content of her university training:

When we studied about research we were doing poster presentations and one of the lecturers said “you know, it is really important to be able to learn how to deliver a presentation because you might go to a conference and you will be presenting something”….and we were all thinking “when are we ever going to go to a conference and present something??”

A number of university students who attended the focus group discussion argued that the university program places an exaggerated emphasis on the teaching of Evidence Based Practice (EBP) and on the principles of scientific research. Such “purely intellectual” subjects were often seen to “steal” valuable time that could otherwise be dedicated to hands-on practice of hands-on medical/practical skills. For example, GA argues that:

The last thing you want to do whilst you’re dealing with a cardiac arrest is start thinking about evidence-based practice…

Indeed, as Evetts (2006) stresses, members of professional groups that are undergoing organizational change, particularly the more experienced, older generation, often perceive reforms as an increase in unnecessary bureaucracy. Furthermore, the change is often seen to interfere with the “routine delivery” of professional work and to “corrupt” the occupational identity that emerges during years of professional socialization at the workplace.

### Second theme: the expansion of paramedic knowledge and skills, professional accountability and the individualization of practice

With the regulation of paramedic practice and the expansion of the paramedic role, greater professional accountability came about. Such accountability can be seen as enhanced professional recognition, status and trust by both managers and clients in paramedics’ ability to perform complex tasks. At the same time, it reflects an externalized form of regulation (Evetts 2013) and a transition from professionalism rooted in practitioners’ moral commitment to professionalism that is driven by managerial control and by practitioners’ fear of being punished if they don’t follow the professional conduct. Following the regulation of paramedic practice, registered paramedics, members of the AHPs, work under clearly defined and strict professional codes of ethics and practice (HCPC 2007). For example, during the period 02/02/2012–10/01/2013, due to lack of compliance with codes of professional practice, 10 registered paramedics were struck off from the HCPC register and 12 paramedics were suspended during that period, by far the highest of all AHPs (Lovegrove and Davis 2013). This in stark contrast to “the old days”, as FM, an experienced paramedic who has been trained during the “pre-reforms” era, recalls:

If I’d done something wrong I’d get a clip around the head, and that was it…

Nowadays, as HK recalls from her first few shifts on the ambulance upon graduating from university,

….you suddenly think “I have to remember absolutely everything they’ve just taught me at university”… And you don’t want to get anything wrong, because that means that your career is gone. You don’t just think about your patient, you constantly think about your own registration.

The last two narratives reflect the tension between occupational and managerial professionalism. Evett’s (2013) describes “occupational professionalism” as a discourse that is constructed from *within* the occupational group and which is distinguished by collegial authority, discretionary decision-making and occupational control, where practitioners are bound by moral commitment and are *trusted* to perform professional tasks. In contrast, the regulation of education and practice, the standardization of expert knowledge, the monitoring of practice through performance indicators (mistrust), the hierarchical nature of the organization and of decision making, all follow a discourse of managerial control (Fournier 1999). The result seems to reflect a gradual decline of the collegial nature of paramedic work towards an *individualization* of practice and the loss of the strong sense of community that characterized the paramedic professional culture. CJ for example, recalls the “old days” prior to the regulation of practice:

There seemed to be a lot more support from your mates. We were much closer. […] Now it is a “you against the world” kind of thing.

Also RC, who is clinical mentor and who has joined the ambulance trust 14 years ago, recalls how different a typical shift used to be when she joined the service:

I remember being back on my station and it was like a family. You knew everyone. You’d sit down on a Sunday morning and all have breakfast together. And you might get “disturbed” for a job but the likelihood is that you’d be kept on base to have your breakfast together and you’d see these people all the time […] Now I think the crew might not see another crew all day.

The study participants, including front-line paramedics, paramedic educators and students, made frequent reference to the “camaraderie”, a spirit of professional friendship that characterized ambulance work and which is obtained during socialization, both while in university and in practice, and is perceived to be fading in recent years. Another commonly-mentioned term that reflects the “pre-reforms” paramedic culture is the “paramedic banter”, a sort of humorous, teasing exchange between colleagues who are also friends or “buddies”. Overall the narratives reflect a sense of loss or yearning to what used to be an inherently communal and socialized occupation but has now been somewhat “domesticated” and individualized in the course of the regulation and monitoring of professional practice.

### Third theme: higher education and professionalism in paramedic practice

A third central theme in participants’ narratives was the nature of the relationship between the “pre-reforms” trained practitioners and the “post-reforms” university graduates. Broadly speaking, this relationship reflects the friction between two discourses of professionalism, organizational (post-reforms education and practice) and occupational (pre-reforms), although this is a rather crude distinction that does not fully reflect the diversity of views amongst paramedics. The development of paramedic science university programs has been driven by the management rather than from within practice (McCann et al. 2013) and as such it can be seen to serve the discourse of organizational professionalism. However, the nature of university education and the socialization that is part of it reflects the spirit of occupational professionalism that is generated from within practice by practitioners themselves (Freidson 2001).

With time, as the volume of university graduating registered paramedics within the ambulance service grew, the complex interaction between the two practitioner groups became more challenging. Moreover, the Paramedic Science university students were often a mixture of “conventional university students”, typically 18-year-old college graduates, and the experienced (and typically older) ambulance technicians commissioned by the ambulance service. As stated by RC, an experienced paramedic educator who is employed by the ambulance service, some of the “pre-reforms” ambulance technicians did not have sufficient academic qualifications to allow them to enroll to the university program, and were therefore unable to become qualified registered paramedics. This often prompted frustration and resentment toward the new graduates:

Some staff would like to get on to paramedic programs and they don’t have the academic qualifications to get on to university. Often that’s one of their bugbears I guess, that they have all this experience but no qualifications to access a university program. Yet, you have a 21-year-old graduate who has the qualifications but no experience and sometimes I think that’s hard for the experienced people to understand and it angers them.

The following two narratives by TJ and RV, both “pre-reforms” ambulance technicians who later completed university paramedic programs, demonstrate the ambiguity that characterizes the view of the new graduates, a mixture of frustration and respect:

TJ: When the university graduates started coming into the service they had so much knowledge, and it bugged me. I was frustrated with that. I felt like I don’t cut it anymore and these guys are so amazing.

RV: The students seem to be coming in with a “higher” plan before they’ve even got their paramedic ticket [..] they do seem to be kind of “dipping in” [to practice] and then they are already thinking of ways to get out.

In the eyes of the veteran, “pre-reforms” trained paramedics, the new university graduates were perceived as both highly knowledgeable yet often lacking the experience, “street-wise” nature and the discretionary decision-making capacity of the “older generation”, as the following narrative by FM, a pre-reforms registered paramedic suggests:

They [university graduates] have not one ounce of common sense, but a lot of knowledge. No life experience with a lot of them…If you give them a blanket they think of it just as a blanket, they don’t realize what you can use a blanket for! Then again, their knowledge of theory is brilliant, they know so much more than we did as paramedics.

The general perception by the pre-reforms trained practitioners was that university graduates who had not been working for the ambulance service prior to their paramedic education do not share the same occupational and collegial commitment and the kind of occupational professionalism that identifies the pre-reforms practitioners. They were portrayed by some of the interviewees to be more self-motivated and, empowered with academic credentials, as primarily motivated by personal gains:

[HP, clinical educator:] You know, I have been in [ambulance] service for over 16 years now. And I will probably stay here. But what we are seeing now is an obvious shift in the workforce. We are getting a lot of university graduates coming in, doing their jobs, getting their university degrees, they are getting some experience under their belt, they stay for 3-4 years and they are moving on.

As for the university graduates, as the narratives suggest, they are having to earn the respect of the “older generation” and demonstrate their “worthiness” as paramedics, first during their university education and later, upon graduation, at the ambulance service as registered paramedics. As students, they are being scrutinized by the academic team as well as, during clinical placement, by the ambulance crew and by the clinical educators. Upon graduation, they have to mold themselves into the paramedic work-culture, while, as registered paramedics, they are the accountable and senior authority. They are put in charge of an ambulance crew and are expected to adhere to professional code of practice and strict performance indicators. This, as SK, the Paramedic Science course leader suggests, is not an easy undertaking for the university graduate:

The students have to have a really thick skin because they are scrutinized. They are scrutinized by us [the academic team] when they are in university. They are scrutinized by registrants when they are in clinical placement. They are scrutinized by patients because they are obviously students and because it is written all over their shirts. So there is a whole host of things that they have to overcome and we are asking 18 year olds to overcome some challenges that a lot of older people would struggle with.

It is now increasingly common that an experienced ambulance crew is being led by a 20-year-old university graduate. Therefore, it is not uncommon that the professional authority of the young paramedic is challenged by a highly experienced, yet lower-ranked, ambulance technician. BJ, who only recently graduated from university, found herself suddenly in charge of an experienced ambulance crew. She recalls her first shift as “traumatic”:

My first ever job was a nightmare. My assistant [an experienced ambulance technician] took decisions that were wrong. But because she has been on the road for a long time I let her make these decisions although I knew they were wrong. It’s difficult to overrule someone when you’ve just been in and she’s been in service for 15 years. […]

Although such encounters are commonly described by the study participants, it seems that with time the “older generation” has also developed a sort of academic appetite. For example, five of the pre-reforms trained interviewees went on to complete undergraduate paramedic education followed by a Master’s degree. TJ, who attended the university program having had no previous academic or college qualifications, describes her academic journey as “empowering”:

To actually go through the process [university education] and end up with a credible mark…I was just blown away. And I can’t tell you what that did for my confidence really. It just empowered me.

Although initially skeptical of university education, when BT finally decided to attend the university program herself, she went on and completed a Master’s degree. Today, she is a lecturer on the BSc (Hons) Paramedic Science program and about to start her PhD:

Obviously, when you get older, you don’t want to be lifting and shifting people. To be honest, having done the paramedic degree, career wise, it really helped me move jobs and plan my future. You are higher up “the food chain”…

## Discussion

Our investigation, although limited in size and in scope, offers primary findings and observations, which call for further studies across the UK and further afield. Participants’ narratives embody and personify the impact of the educational reforms on paramedics’ professional identity and professional culture while illuminating central concepts in the study of the relationship between higher education and professional practice in health and social care.

According to the study of professionalization and occupational closure, HEIs provide “classic” professions such as medicine with the certification mechanism to determine professional membership that is based on the acquisition of expert knowledge, as well as the space to monitor the specialization and codification of complex expert knowledge by defining educational standards and by providing training programs that are supported by the state (Abbott 1988; Freidson 1986; MacDonald 1995). HEIs, in that respect, are enablers of occupational closure and of professional jurisdiction by distinguishing between those who are professionals (and who are able to join the professional association) and those who are non-professionals. Nevertheless, as the case of paramedic education in the UK demonstrates, the picture is far more complex.

First, it is difficult to use the perspective of occupational closure to newly-emerging occupations (such as paramedics) which are not equipped with the same societal and political standing of “elite professions” such as medicine (Evetts 2013; Noordegraaf 2007). In fact, studies have demonstrated how the medical profession exercise control over the delivery of expert knowledge in the health care domain (Freidson 1986; Larkin 1980; Saks 2012) and the subordination of AHPs to the medical profession in both practice and education (Cant et al. 2011; Nancarrow and Borthwick 2005; Witz 1990). Indeed, as argued by McCann et al. (2013), due to its “top-down” nature and without engaging paramedics “on the ground” in the occupational change, the professionalization of British paramedics and the formalization of paramedic education did not quite grasp practitioners’ support and it did not bear the professional gains that are expected as part of occupational closure such as better pay or better work conditions.

Second, the process of “being a professional” goes beyond the obtaining of certificates and formal qualifications through educational programs. It goes also beyond ‘professional behavior’ that is defined by standard codes of ethics and practice that are developed by regulators and professional bodies (Morrow et al. 2011). Professionalism is also developed as *an identity*, perceived and enacted, while being part of a professional community and, as such, it is also constructed through socialization (Evetts 2013). Universities offer a space for paramedics’ socialization during the students’ shared academic journey and while in clinical placement. Universities also provide a space for institutionalized professional community through academic conferences, academic profession-related journals and research programs (Noordegraaf 2007). Since the emergence of paramedic education in universities there has been a notable development of paramedical academic activities. *The Journal of Paramedic Practice* publishes a mixture of clinical and professional reports as well as research publications, and paramedic and emergency care research conferences are organized by universities and by the College of Paramedics, bringing the professional community together while expanding the boundaries of professional practice. Table 1, below, is a summary of the influence of the formalization of paramedic education in HEIs on the professionalization of paramedics:

**Table 1:** A summary of participants' perceived professional related influences of paramedic university education

Front-line paramedics	Enhanced public health roles and with it enhanced accountability and public trust Individualized practice and decreased sense of community and ‘camaraderie’ Cultural tension between the ‘pre-reforms’ trained paramedics and the university graduates
Paramedic students	Formal education, certificates, enhanced societal status University and clinical placement socialization Sense of achievement, professional pride, public recognition Potential for greater job mobility Interaction with the academic community and with other professional groups Intensified scrutiny as an academic discipline
The ambulance service	Injection of university-trained, knowledgeable workforce equipped with enhanced medical knowledge and skills Greater job mobility may lead to challenges in retaining paramedics
The host university	Struggle to locate itself between academic and professional requirements Struggle to locate itself between occupational and organizational professioanlism

As can be seen in Table 1, participants’ narratives reflect the complex and somewhat paradoxical role of higher education in shaping professionalism in paramedic practice. The university, now the provider of paramedic education, finds itself in between the organizational professionalism that is driven by the ambulance service management and the occupational professionalism that is led, from within practice, by front-line paramedics and university students. What then is the role of the host university as part of the professional transformation? For the “pre-reforms”, front-line paramedics, the expansion of paramedic knowledge and of the paramedic role offer greater responsibility, accountability and job-complexity. However, it is being linked with the loss of the communal occupational nature of paramedic practice.

The “new paramedics”, the university graduates, are awarded with academic certificates, placing them amongst other established academic-professional health care professionals, possibly providing them with greater job mobility. At the same time, they are under intense scrutiny, first as students and as ‘newcomers’ to the ambulance service, and later, like their ‘pre-reforms’ peers, working under strict performance indicators and professional reviews. Therefore, although the university enables the expansion and the medicalization of expert knowledge and with it the enhancement of the paramedic public health role, it is linked also with organizational control of the individual practitioner and the challenge to paramedics' professional autonomy and discretionary decision-making.

Like other AHPs, professionalism is inherent to paramedic education and code of practice. As part of the program, students on the BSc (Hons) Paramedic Science at the host university engage with professionalism which underlines ‘professional behavior’, based on ‘ethical’, ‘reliable’ and ‘effective’ practice (Morrow et al. 2011). At the same time, the examination of the role of HEIs in shaping paramedics’ professional identity reflects the increasingly ambiguous nature of professionalism in AHPs’ practice. The meaning of professionalism is not fixed (Evetts 2003, p. 407), and different interpretations of professionalism are needed in order to understand its appeal to AHPs, and with it, understand the role of universities in facilitating occupational change.

## Footnotes

1 Emergency Medical Technician (EMT) or Ambulance technician is a clinical role in the UK ambulance services but is unregistered. They are trained to a lower standard than paramedics.
